# Prevalence and characteristics of comorbid stroke and traumatic brain injury in a real-world population: findings from a nationally representative cross-sectional survey in China

**DOI:** 10.1186/s12889-023-16990-0

**Published:** 2023-10-18

**Authors:** Bin Jiang, Dongling Sun, Haixin Sun, Xiaojuan Ru, Hongmei Liu, Siqi Ge, Jie Fu, Wenzhi Wang

**Affiliations:** 1 Department of Neuroepidemiology, Beijing Neurosurgical Institute, Beijing Tiantan Hospital, Capital Medical University, No. 119, South Fourth Ring Road West, Fengtai District, Beijing, 100070 P. R. China; 2 Beijing Municipal Key Laboratory of Clinical Epidemiology, Beijing, China; 3 National Office for Cerebrovascular Diseases (CVD) Prevention and Control in China, Beijing, China

**Keywords:** Stroke, Traumatic brain injury, Comorbidity, Prevalence, Population

## Abstract

**Background:**

In China, data on the prevalence and characteristics of comorbid stroke and traumatic brain injury (TBI) in real-world populations are still lacking but of paramount importance for the evidence-based prevention and control of the comorbidity of the two diseases. This study aimed to investigate the prevalence and characteristics of comorbid stroke and TBI in a real-world population.

**Methods:**

In 2013, a nationally representative, door-to-door survey on stroke and TBI using a complex, multistage, probability sampling design was conducted among approximately 600,000 participants from 155 urban and rural areas in China (Ethic ID: KY2013-006-01). The weighted prevalence of comorbid stroke and TBI was estimated using individuals’ final weight. A Poisson regression analysis was used to compare the rate ratio of the comorbidity prevalence among different subgroups of the population, including age, sex, place of residence, and geographic location subgroups. For analyses of associations between the comorbidities and predictors of interest, all other variables were adjusted for in a multinomial logistic regression model.

**Results:**

Among the 596,536 people, 219 patients with comorbid stroke and TBI were identified. The point prevalence of comorbid stroke and TBI weighted to the China 2010 census population was 29.30 (95% CI: 22.69–37.84) per 100,000 population in China. The adjusted prevalence of post-TBI stroke in patients with previous TBI was significantly higher than that of post-stroke TBI in patients with previous stroke (6021.3 vs. 811.1 per 100,000 people; rate ratio: 11.001; 95% CI: 8.069–14.998). Patients with nonconcussion had significantly higher rates of both pre-stroke TBI (odds ratio: 4.694; 95% CI: 3.296–6.687) and post-stroke TBI (odds ratio: 6.735; 95% CI: 3.719–12.194) than patients with concussion. Compared to patients with ischaemic stroke, patients with subarachnoid haemorrhage (odds ratio: 2.044; 95% CI: 1.097–3.809) and intracerebral haemorrhage (odds ratio: 1.903; 95% CI: 1.296–2.795) had significantly higher rates of post-TBI stroke.

**Conclusions:**

The high prevalence of stroke among TBI patients is becoming a new public health issue. TBI patients, especially those with nonconcussion TBI, are more likely to develop comorbid stroke and TBI than stroke patients, especially ischaemic stroke patients.

**Supplementary Information:**

The online version contains supplementary material available at 10.1186/s12889-023-16990-0.

## Introduction

According to a series of studies on the global burden of disease, China has a higher burden of both stroke [1, 2] and traumatic brain injury (TBI) [3] than other countries or regions of the world. A nationally representative cross-sectional survey conducted in China in 2013 estimated that 1114.8 per 100,000 people aged 20 years and older in the population had experienced a stroke [4], and 442.4 per 100,000 people of all ages had experienced a TBI [5]. Globally, the age-standardized prevalence of stroke was 1240.3 per 100,000 people in 2019 [2], and the age-standardized prevalence of TBI was 759.0 per 100,000 people in 2016 [3]. The prevalence of stroke increased by 22% among people younger than 70 years worldwide from 1990 to 2019 [2], whereas the prevalence of TBI increased by 8.4% from 1990 to 2016 [3]. The prevalence of stroke increased from 364.5 to 100,000 people in 1985 to 929.9 per 100,000 people in 2013 in rural areas of China and from 667.9 to 100,000 people to 789.4 per 100,000 people in urban areas of China. [4] The increasing prevalence of stroke and TBI in the population in recent years has greatly increased the probability and harm of the comorbidity of the two diseases. TBI is an independent risk factor for stroke, regardless of TBI severity or type, which has been further confirmed by previous meta-analyses [6, 7]. To date, only one study [8] has explored the impact of previous stroke on TBI, in addition to previous studies on post-stroke falls. More research suggests that stroke and TBI increase each other’s risk. It also increases the risk of comorbid stroke and TBI.

In China, a cohort study from Taiwan Province first confirmed that brain trauma increases the risk of stroke in 2011 [9]; however, the prevention and control of stroke following TBI have not received sufficient attention. An accurate estimate of the prevalence and characteristics of comorbid stroke and TBI in a real-world population is essential to reliably inform prevention and control strategies, understand population distributions, and ultimately prevent comorbid stroke and TBI. However, evidence on the prevalence and characteristics of comorbid stroke and TBI in a real-world population is still lacking in China. Therefore, this study aimed to analyse the prevalence and characteristics of comorbid stroke and TBI in a real-world population using a nationally representative cross-sectional survey of stroke and TBI in China [4, 5].

## Methods

### Study design

A nationally representative cross-sectional sampling survey on stroke and TBI was conducted in mainland China from September 1 to December 31, 2013, which was described in detail in previous studies [4, 5, 10, 11]. A post hoc analysis of the comorbidity of the two diseases was performed in this study.

### Study setting

**Fig. 1:**
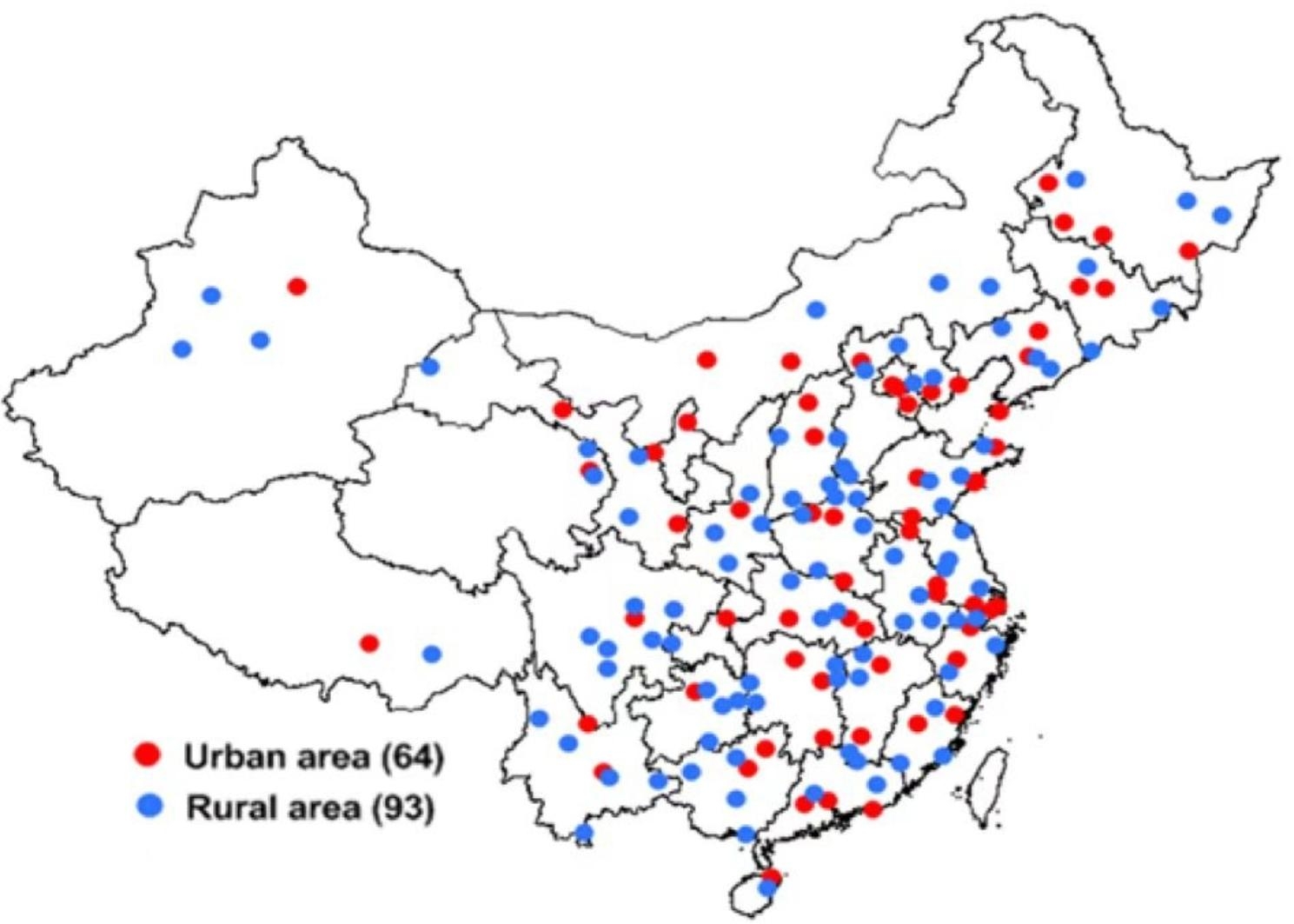
Distribution of survey sites in 31 provinces of China

A total of 64 urban and 93 rural areas from 31 provinces of China, which were determined to be used for disease surveillance in China, were defined as the sampling frame for this survey. The survey was carried out at 157 disease surveillance points [see Fig. 1].

### Eligibility criteria for participants

The participants included people who had lived in the defined areas for at least 6 months in the year before the survey was conducted.

### Sampling technique and sample size

A complex, multistage probability sampling design was used to define the sampling frame and the participants; namely, 2010 Chinese population census data and probability proportionate to population size (PPS) sampling were used to select 64 urban and 93 rural areas from 31 provinces of China. The sampling frame for this survey was the National Disease Surveillance Points (DSP) System with a population of approximately 74 million people which may represent the 2010 China national census data concerning geographical distribution, social status, and economic status. In the first stage of sampling, PPS sampling was again used to select “neighbourhoods” (Jiedao) within cities or “townships” (Xiang) in rural areas; the probability of selection was based on the population size of the neighbourhood or township. In the second stage of sampling, one or more neighbourhood committees (administrative villages) with a total population of at least 4,500 residents (~ 1,500 households) were selected from the sampled neighbourhood (townships) at each site using random cluster sampling. The sample size for this survey was calculated at approximately 600,000 subjects based on a 1% stroke prevalence, with a two-sided 95% CI, a design effect (deff) value of 5, and a relative error of 15%, according to the complex sampling formula as follows:

$$N=deff\frac{{u}^{2}p(1-p)}{{d}^{2}}$$

### Data collection, case ascertainment, and quality assurance

In this survey, participants with suspected stroke symptoms and a self-reported history of traumatic brain or spinal cord injury were identified by CDC investigators and interviewed by neurologists. From 1 September through 31 December 2013, CDC investigators visited each household in the sample population to collect the participants’ signed informed consent forms and complete the preliminary screening form, including basic information about family members, family deaths since 1 January 2012, and positive stroke symptoms and self-reported history of traumatic brain or spinal cord injuries experienced by family members. Participants who had positive stroke symptoms and a self-reported history of traumatic brain or spinal cord injury were invited to see a neurologist in a town/village clinic. Diagnoses of definite and probable stroke or traumatic brain or spinal cord injury were determined after the neurologist completed a physical examination and reviewed previous medical documents, including an identity card or household register, the medical history of the individual with stroke or a traumatic brain or spinal cord injury, and computed tomography (CT)/magnetic resonance imaging (MRI) scans.

Quality control was involved in all phases of the survey, and survey preparations, fieldwork, and data processing were all supervised. [4, 5, 10, 11] Trained investigators visited these participants at least 3 times on different dates to ensure a response. Three tiers of a quality control network separately formed by CDC investigators and hospital neurologists, i.e., national, provincial, and local quality control groups, were established to supervise investigators, the organization and mobilization of the survey, and questionnaire inquiries. All of the questionnaires were mailed to the Beijing Neurosurgical Institute at the end of the investigation, checked by research staff, and then entered twice into a database by specialized staff according to a strict procedure. In this survey, two of the 157 DSPs were excluded from the final data analysis due to not meeting the requirements of the study design. For the prevalence analysis in this survey, 596,536 individuals from 178,059 families were ultimately included, with a response rate of approximately 81% [10, 11].

### Diagnostic criteria

In this epidemiological study, the minimum criterion for definite or probable stroke was evidence of a sudden or rapid onset of neurological symptoms lasting for > 24 h or leading to death without evidence of a nonstroke cause [4, 10]. Any patients with nervous system abnormalities induced by trauma, metabolic disorders, tumours, or central nervous system infections were excluded. Whenever brain imaging was used within the first week of stroke onset and the imaging results were available for review by the study neurologist, the pathological type of stroke was classified into 4 major categories: subarachnoid haemorrhage (SAH; only lumbar puncture was allowed for the diagnosis of SAH); intracerebral haemorrhage (ICH); and ischaemic stroke (IS). ; and stroke of an undetermined pathological type in the case of stroke patients with no brain imaging performed within the first week of stroke onset or when the imaging results were not available for review by the study neurologist.

TBI is commonly defined as an alteration in brain function or other evidence of brain pathology caused by an external force based on the Common Data Elements definition [5]. In the present survey, TBIs were assessed by asking the participants if they had ever suffered from a physician-diagnosed TBI that had resulted in a cerebral concussion or more severe head injury and further confirmed by trained investigators using a structured questionnaire. Accordingly, TBI was roughly divided into concussion and nonconcussion TBI, including cerebral contusion and laceration, traumatic intracranial haematoma, brain stem injury, traumatic brain and spinal cord injuries, and unclassified TBI.

Self-reported information on age, sex, ethnicity, education level, marital status, and current occupation, as well as the medical history of the individuals with stroke [4] and data on times and dates, sites, symptoms and signs, external causes, and medical treatments for TBI [5] and/or spinal cord injury [11] were also obtained and reviewed. In this survey, each target event of stroke and TBI within a lifetime was recorded carefully for all the participants. Hypertension was defined as having a history of hypertension or taking antihypertensive medication in the last 4 weeks, having a systolic blood pressure ≥ 140 mmHg, or having a diastolic blood pressure ≥ 90 mmHg. A history of diabetes mellitus was defined by patient self-reports of having been told by a doctor that they had diabetes mellitus or using antidiabetic drugs. Atrial fibrillation was diagnosed by ECG, as per medical records. The diagnosis of coronary heart disease (CHD) included a history of myocardial infarction or angina documented in medical records. Dyslipidaemia was diagnosed by medical records. Current smoking (≥ 1 cigarette per day) and alcohol intake (any dose of alcohol, ≥ 1 time per week) were defined by the subjects’ self-reports.

### Variables

The characteristics of the study sample included age group, sex, ethnicity, education level, marital status, occupation type, place of residence, and geographic location. The primary outcome was the lifetime point prevalence of comorbid stroke and TBI, which was defined as the rate of patients with stroke and TBI before midnight on August 31, 2013, among the survivors from the sampled families. A Poisson regression analysis was used to compare the rate ratio of the comorbidity prevalence of stroke and TBI among different subgroups of the population, including age, sex, place of residence, and geographic location subgroups. The secondary outcomes were the different comorbidities with first-ever stroke following first-ever TBI (i.e., post-TBI stroke or pre-stroke TBI) and with first-ever TBI following first-ever stroke (i.e., post-stroke TBI or pre-TBI stroke). The analysis of comorbidity for TBI patients involved variables including TBI diagnosis and the external cause of injury in addition to characteristic variables; the analysis of comorbidity for stroke patients involved variables including the subtype of first-ever stroke, disease histories of hypertension, diabetes mellitus, dyslipidaemia, atrial fibrillation, and coronary heart disease, smoking and alcohol consumption.

#### Errors and bias

In this survey, to control sampling errors and response bias, we adopted cluster sampling and did not allow the sampled participants to be replaced; we ensured a response rate of over 80%; and eliminated data from 2 survey points due to not meeting the requirements of the study design.

To ensure the representativeness of the sampling survey, we considered sampling weights, nonresponse weights, and poststratification weights in the complex sampling designs during analysis.

For analyses of rate ratios or associations between each outcome and the predictors of interest, all other variables were adjusted for in a Poisson regression model or a multinomial logistic regression model. However, for the analysis of the rate ratio, we only consider age group, sex, place of residence, and geographic location due to these factors being considered in the sample size estimate.

### Statistical analysis

The sociodemographic characteristics of the study sample were categorized and are presented as numbers and crude and weighted rates. In this retrospective epidemiological survey, the point prevalence of comorbid stroke and TBI was defined as the rate of patients with stroke and TBI before midnight on August 31, 2013, among the survivors from the sampled families. The crude point prevalence of comorbid stroke and TBI was calculated by subgroups of age (0 to 14/15 to 24/25 to 34/35 to 44/45 to 54/55 to 64/65 to74/75 to 84/≥85 years), sex (male/female), place of residence (urban/rural), and geographic location (eastern/central/western China). Weighted coefficients were calculated by considering sampling weights, nonresponse weights, and poststratification weights in the complex sampling designs. Population information from the 2010 China census data was used to calculate poststratification weights [10]. The weighted prevalence of comorbid stroke and TBI was estimated using individuals’ final weights to obtain national estimates. For comparison, the prevalence of overall age groups was directly standardized to the age distribution of the WHO world standard population. The 95% CIs for all the crude, weighted, and age-standardized rates were also calculated. Prevalent numbers of comorbid cases of stroke and TBI in China nationwide were estimated based on the weighted rates of the Chinese census population in 2010.

A Poisson regression analysis was used to compare the rate ratio of the prevalence of comorbid stroke and TBI among different subgroups of the population in China in 2013. Age group, sex, place of residence, and geographic location were introduced in the Poisson regression analyses. For each predictor of interest, all other variables were adjusted for in a Poisson regression model. Previous histories of TBI and stroke in individuals were confirmed and compared by the dates of first-ever TBI and stroke. Furthermore, the rate ratio of the prevalence of post-TBI stroke among TBI patients versus the prevalence of poststroke TBI among stroke patients in the population was also analysed by adjusting for age group, sex, place of residence, and geographic location in a Poisson regression analysis.

Factors associated with pre-stroke TBI and post-stroke TBI using isolated TBI as a reference (pre-stroke TBI/post-stroke TBI/isolated TBI) or factors associated with pre-TBI stroke and post-TBI stroke using isolated stroke as a reference (pre-TBI stroke/post-TBI stroke/isolated stroke) were separately determined. For each predictor of interest, all other explanatory risk factors were adjusted for in a multinomial logistic regression model. The explanatory risk factors included age group (< 35/35 to 44/45 to 54/55 to 64/65 to 74/75 to 84/≥85 years), sex (male/female), ethnicity (Han ethnicity/other ethnicity), education level (primary school and preschool/middle school/college and higher), marital status (married/single/widowed/other), occupation type (farmer/worker/employee/retiree or homemaker/other), geographic location (eastern/central/western China), place of residence (urban/rural), TBI diagnosis (nonconcussion TBI/concussion), external cause (motor vehicle collision/fall/strike/other) for TBI patients and stroke subtype (ischaemic stroke/intracerebral haemorrhage/subarachnoid haemorrhage/unclassified stroke), hypertension (yes/no), histories (yes/no/unknown) of diabetes mellitus, dyslipidaemia, atrial fibrillation and coronary heart disease, smoking status (regular smoking/occasional smoking/quit smoking/never smoked/unknown), and alcohol consumption (regular alcohol consumption/occasional alcohol consumption/stopped consuming alcohol/never consumed alcohol/unknown) for stroke patients.

All statistical calculations on complex samples were performed using IBM® SPSS® statistics for Windows version 21.0 (IBM Corp, Armonk, NY, USA) or SAS® software version 9.4 (SAS Institute Inc., Cary, NC, USA). P < 0.05 was considered statistically significant.

## Results

The characteristics of the study sample from the national epidemiological survey of comorbid stroke and TBI in China in 2013 are shown in Table 1. Among the 596,536 people, there were 7679 surviving first-ever stroke patients, including 5967 ischaemic stroke patients, 1218 intracerebral haemorrhage patients, 338 subarachnoid haemorrhage patients, and 156 unclassified stroke patients. The crude and weighted stroke prevalences were 1287.3/100,000 and 844.5/100,000, respectively.

**Table 1:** Characteristics of the study sample from the national epidemiological survey of comorbid stroke and traumatic brain injury (TBI) in China, 2013

Characteristics	Population	
	No. (Rates)	Weighted rates^†^ (95% CI)
Age group, n (%)		
0–14	83,028(13.9%)	16.8%(15.6-18.0%)
15–24	77,354 (13.0%)	17.1% (16.3-17.9%)
25–34	91,435 (15.3%)	14.9% (14.2-15.5%)
35–44	99,582 (16.7%)	18.3% (17.6-19.0%)
45–54	93,763 (15.7%)	13.9% (13.4-14.5%)
55–64	80,155 (13.4%)	10.5% (10.0-11.1%)
65–74	44,840 (7.5%)	5.5% (5.1-5.8%)
75–84	22,200 (3.7%)	2.6% (2.5-2.8%)
≥ 85	4179 (0.7%)	0.5% (0.4-0.5%)
Sex, n (%)		
Male	300,192 (50.3%)	51.1% (50.8-51.5%)
Female	296,344 (49.7%)	48.9% (48.5-49.2%)
Ethnicity, n (%)		
Han	521,343 (87.4%)	91.8% (88.6-94.1%)
Other	75,193 (12.6%)	8.2% (5.9-11.4%)
Education, n (%)		
Primary school	248,916 (41.7%)	39.6% (35.9-43.4%)
Middle school	294,209 (49.3%)	49.0% (46.5-51.5%)
College and higher	51,730 (8.7%)	11.2% (9.0-14.0%)
Unknown	1681 (0.3%)	0.19% (0.16-0.23%)
Marital status, n (%)		
Married	391,160 (65.6%)	60.9% (59.9-61.9%)
Single	116,817 (19.6%)	24.0% (22.3-25.8%)
Widowed	32,734 (5.5%)	4.2% (3.8-4.6%)
other	53,735 (9.0%)	10.6% (8.9-12.6%)
Unknown	2090 (0.4%)	0.25% (0.21-0.30%)
Occupation, n (%)		
Student	108,978 (18.3%)	23.1% (21.7-24.6%)
Worker	45,021 (7.5%)	8.7% (7.1-10.8%)
Farmer	271,068 (45.4%)	38.6% (33.5-44.0%)
Employee	46,676 (7.8%)	9.8% (7.5-12.7%)
Entrepreneur	52,518 (8.8%)	10.2% (8.0-12.9%)
Retiree or homemaker	66,169 (11.1%)	8.7% (6.8-11.2%)
other	4439 (0.7%)	0.70% (0.51-0.97%)
Unknown	1667 (0.3%)	0.20% (0.16-0.24%)
Place of residence, n (%)		
Urban	282,945 (47.4%)	52.9% (44.3-61.3%)
Rural	313,591 (52.6%)	47.1% (38.7-55.7%)
Geographic location, n (%)		
Eastern China	201,354(33.8%)	40.7%(32.9-49.1%)
Central China	239,735(40.2%)	32.0%(24.4-40.7%)
Western China	155,447(26.1%)	27.3%(19.7-36.5%)
Estimated prevalence (1 per 100,000)		
Stroke	7679(1287.3/100,000)	844.5/100,000(800.0/100,000 − 941.2/100,000)
TBI	2685(450.1/100,000)	434.9/100,000(346.3/100,000 − 546.1/100,000)

†, complex sample weights were used to obtain nationally representative estimates

Among the 596,536 people, there were 2685 surviving first-ever TBI patients, including 1785 concussion patients and 900 nonconcussion TBI patients. The nonconcussion TBI patients included 251 patients with cerebral contusions and lacerations, 337 patients with traumatic intracranial haematomas, 32 patients with brain stem injuries, 36 patients with traumatic brain and spinal cord injuries, and 244 patients with unclassified TBIs. The crude and weighted TBI prevalences were 450.1/100,000 and 434.9/100,000, respectively.

Among the 596,536 people, 219 surviving patients with comorbid stroke and TBI were identified on August 31, 2013 (see Tables 1 and 2). Among the 219 patients, there were 158 patients with first-ever stroke following first-ever TBI (i.e., post-TBI stroke or pre-stroke TBI) and 61 patients with first-ever TBI following first-ever stroke (i.e., post-stroke TBI or pre-TBI stroke). The median time to stroke onset post-TBI was 11.07 (IQR: 2.97–26.98) years among the 158 patients with first-ever stroke following first-ever TBI, whereas the median time to TBI onset post-stroke was 3.00 (IQR: 0.99–7.03) years among the 61 patients with first-ever TBI following first-ever stroke.

**Table 2:** Prevalence* of comorbid stroke and traumatic brain injury in the Chinese population (1/100,000 person*lifetime)

Age group	Male				Female				Total	
	Population	cases	Prevalence^†^	95% CI^†^	Population	cases	Prevalence^†^	95% CI^†^	Prevalence^†^	95% CI^†^
0~	44,364	0	0.00	-	38,664	0	0.00	-	0.00	-
15~	39,589	1	2.53	0.06–14.07	37,765	0	0.00	-	1.29	0.03–7.20
25~	45,020	1	2.22	0.06–12.38	46,415	1	2.15	0.05-12.00	2.19	0.26–7.90
35~	50,759	5	9.85	3.20-22.99	48,823	4	8.19	2.23–20.98	9.04	4.13–17.16
45~	46,879	25	53.33	31.87–72.71	46,884	9	19.20	8.78–36.44	36.26	25.11–50.67
55~	39,366	43	109.23	79.05-147.13	40,789	19	46.58	28.04–72.74	77.35	59.30-99.16
65~	21,902	51	232.86	173.38-306.16	22,938	27	117.71	77.57-171.26	173.95	137.50-217.10
75~	10,545	22	208.63	130.75-315.87	11,655	8	68.64	29.63-135.25	135.14	91.18-192.91
≥ 85	1768	2	113.12	13.70-408.64	2411	1	41.48	1.05-231.09	71.79	14.80-209.79
Total	300,192	150	49.97	42.29–58.63	296,344	69	23.28	18.12–29.47	36.71	32.01–41.91
Age-adjusted rates^‡^	-	-	35.05	29.63–41.63	-	-	15.51	12.06–20.33	25.06	21.83–28.88
Weighted rates^§^	-	-	41.73	30.81–56.52	-	-	16.29	11.69–22.69	29.30	22.69–37.84
Prevalent No.	-	-	279597.47	206423.85-378678.22	-	-	104278.31	74843.44-145269.27	383875.79	297289.95-495788.96

†, a point prevalence in a lifetime, on 31 Aug, 2013

‡, standardized to WHO world standard population

§, weighted to China census population 2010

### Prevalence of comorbid Stroke and TBI

In China, the point prevalences of comorbid stroke and TBI weighted to the China 2010 census population were as follows: 29.30 (95% CI: 22.69–37.84) per 100,000 population; 41.73 (95% CI: 30.81–56.52) per 100,000 men; 16.29 (95% CI: 11.69–22.69) per 100,000 women (see Table 2); 31.77 (95% CI: 21.45–47.07) per 100,000 urban residents; 26.52 (95% CI: 19.99–35.18) per 100,000 rural residents; 27.81 (95% CI: 16.04–48.23) per 100,000 eastern China residents; 32.86 (95% CI: 26.00–41.52) per 100,000 central China residents; and 27.34 (95% CI: 17.47–42.77) per 100,000 western China residents. According to the above-estimated prevalences, there were an estimated 383,876 (95% CI: 197,290–495,789) patients with comorbid stroke and TBI in the population in China, with 279,597 (95% CI: 206,424–378,678) male patients and 104,278 (95% CI: 74,843–145,269) female patients.

After adjusting for other factors, including sex, place of residence, and geographic location, the prevalence of comorbid stroke and TBI increased with age up to the age group of 75 years or older. The prevalence of comorbid stroke and TBI among men was significantly higher than that among women (rate ratio: 2.273; 95% CI: 1.709–3.023). No difference in the prevalence of comorbid stroke and TBI was found between residents in urban and rural areas and eastern, central, and western China (see Table 3).

**Table 3:** Prevalence (1/100,000 person*lifetime) and rate ratio of comorbid stroke and traumatic brain injury among different subgroups of the Chinese population

Factors	prevalence		
	Rate (95%CI)	Rate ratio^†^ (95%CI)	P value
Age group			
0~			
15~	1.29(0.03–7.20)	0.016(0.002–0.156)	< 0.001
25~	2.19(0.26–7.90)	0.028(0.005–0.166)	< 0.001
35~	9.04(4.13–17.16)	0.113(0.031–0.418)	0.001
45~	36.26(25.11–50.67)	0.461(0.142–1.502)	0.199
55~	77.35(59.30-99.16)	0.999(0.314–3.184)	0.999
65~	173.95(137.50-217.10)	2.218(0.700-7.029)	0.176
75~	135.14(91.18-192.91)	1.757(0.536–5.759)	0.352
85~	71.79(14.80-209.79)	Reference	
Sex			
Male	49.97(42.29–58.63)	2.273(1.709–3.023)	< 0.001
Female	23.28(18.12–29.47)	Reference	
Place of residence			
Urban	40.64(33.55–48.79)	1.093(0.836–1.428)	0.516
Rural	33.16(27.10-40.18)	Reference	
Geographic Location			
Eastern China	28.80(21.87–37.24)	0.738(0.506–1.076)	0.114
Central China	45.88(37.71–55.30)	1.243(0.890–1.737)	0.202
Western China	32.81(24.43–43.14)	Reference	

†, for each predictor of interest, all other variables in the table were adjusted in a Poisson regression model

### Higher prevalence of post-TBI stroke among persons with TBI versus a lower prevalence of post-stroke TBI among patients with stroke in the population

The prevalence of post-TBI stroke was 6021.3 per 100,000 people among 2624 persons with TBI, whereas the prevalence of post-stroke TBI was 811.1 per 100,000 people among 7521 patients with stroke. After adjusting for other factors, including age group, sex, place of residence, and geographic location, the prevalence of post-TBI stroke in individuals with previous TBI was significantly higher than the prevalence of post-stroke TBI in individuals with previous stroke (rate ratio: 11.001; 95% CI: 8.069–14.998; P < 0.001).

### Factors associated with pre-stroke TBI and post-stroke TBI using isolated TBI as a reference

Among the TBI patients, the rates of both pre-stroke TBI (odds ratio: 0.075; 95% CI: 0.008–0.698) and post-stroke TBI (odds ratio: 0.014; 95% CI: <0.001–0.445) in the age group younger than 35 years were significantly lower than those in the age group aged 85 years or older. Only men had a significantly higher rate of pre-stroke TBI than women (odds ratio: 1.751; 95% CI: 1.170–2.622), but no significant sex difference in the rate of post-stroke TBI was found. Compared to farmers, retirees and homemakers had a significantly higher rate of pre-stroke TBI (odds ratio: 1.982; 95% CI: 1.271–3.091), but no significant difference in the rate of post-stroke TBI was found. In contrast, residents of eastern (odds ratio: 2.509; 95% CI: 1.063–6.353) and central (odds ratio: 4.237; 95% CI: 1.899–9.456) China had a significantly higher rate of post-stroke TBI than those of western China, but no significant difference in the rate of pre-stroke TBI was found. Compared to TBI patients in the concussion group, TBI patients in the nonconcussion group had significantly higher rates of both pre-stroke TBI (odds ratio: 4.694; 95% CI: 3.296–6.687) and post-stroke TBI (odds ratio: 6.735; 95% CI: 3.719–12.194) (see Table 4).

**Table 4:** Factors associated with pre-stroke traumatic brain injury (TBI) and post-stroke TBI using isolated TBI as a reference

Factors	Pre-stroke TBI			Post-stroke TBI		
	Adjusted^†^ OR	95%CI	P value	Adjusted^†^ OR	95%CI	P value
Age group						
< 45	0.202	0.081–0.503	0.001	0.051	0.010–0.255	< 0.001
45–54	0.586	0.291–1.182	0.136	0.210	0.074–0.595	0.003
55–64	0.875	0.473–1.621	0.672	0.313	0.132–0.743	0.008
65–74	1.677	0.937–3.003	0.082	0.843	0.391–1.819	0.663
≥ 75	Reference	Reference		Reference	Reference	
Sex						
Male	1.750	1.169–2.619	0.007	0.741	0.416–1.319	0.308
Female	Reference	Reference		Reference	Reference	
Ethnicity						
Han ethnicity	1.099	0.561–2.154	0.783	2.513	0.577–10.939	0.220
Other	Reference	Reference		Reference	Reference	
Education						
Primary school and preschool	1.879	0.623–5.668	0.263	1.003	0.209–4.824	0.997
Middle school	2.111	0.707-6.300	0.180	1.350	0.279–6.519	0.709
College and higher	Reference	Reference		Reference	Reference	
Marital status						
Single	0.224	0.029–1.703	0.148	0.691	0.082–5.822	0.734
Widowed	1.059	0.644–1.740	0.822	0.458	0.195–1.074	0.072
Other	0.660	0.056–7.733	0.740	2.971	0.330-26.716	0.331
Married	Reference	Reference		Reference	Reference	
Occupation						
Worker	1.015	0.510–2.019	0.967	0.409	0.091–1.842	0.244
Employee	1.061	0.395–2.845	0.907	0.512	0.065–4.009	0.524
Retiree or homemaker	1.973	1.266–3.075	0.003	1.116	0.552–2.255	0.759
Other	0.998	0.174–5.713	0.998	1.404	0.187–10.542	0.741
Farmer	Reference	Reference		Reference	Reference	
Geographic location						
Eastern China	1.319	0.822–2.117	0.251	2.586	1.061–6.306	0.037
Central China	1.494	0.983–2.270	0.060	4.161	1.872–9.249	< 0.001
Western China	Reference	Reference		Reference	Reference	
Place of residence						
Urban	0.957	0.650–1.410	0.825	0.735	0.401–1.348	0.320
Rural	Reference	Reference		Reference	Reference	
TBI diagnosis						
Non-concussion TBI	4.685	3.289–6.673	< 0.001	6.663	3.680-12.063	< 0.001
Cerebral concussion	Reference	Reference		Reference	Reference	
External cause						
Motor vehicle collision	0.495	0.195–1.255	0.138	0.431	0.116-1.600	0.209
Fall	1.163	0.478–2.833	0.739	0.793	0.225–2.790	0.717
Strike	0.991	0.386–2.545	0.984	0.556	0.136–2.266	0.413
Other	Reference	Reference		Reference	Reference	

†, for each predictor of interest, all other variables in the table were adjusted in a multinomial Logistic regression model

### Factors associated with pre-TBI Stroke and post-TBI Stroke using isolated Stroke as a reference

Among the stroke patients, the rate of post-TBI stroke (odds ratio: 5.183; 95% CI: 1.011–26.566) in the age group older than 35 to 44 years was significantly higher than that in the age group aged 85 years or older. Men had a significantly higher rate of post-TBI stroke than women (odds ratio: 1.976; 95% CI: 1.261–3.097). In contrast, residents of eastern (odds ratio: 0.514; 95% CI: 0.328–0.804) and central (odds ratio: 0.538; 95% CI: 0.360–0.806) China had a significantly lower rate of post-TBI stroke than those in western China. Compared to IS patients, SAH (odds ratio: 2.044; 95% CI: 1.097–3.809) and ICH patients (odds ratio: 1.903; 95% CI: 1.296–2.795) had significantly higher rates of post-TBI stroke. However, no factor significantly associated with comorbid pre-TBI stroke was found. No difference in the rate of pre-TBI stroke or post-TBI stroke among patients with stroke was found between the two groups with and without stroke-related factors (see Table 5).

**Table 5:** Factors associated with pre-traumatic brain injury (TBI) stroke and post-TBI stroke using isolated stroke as a reference

Factors	Pre-TBI stroke			Post-TBI stroke		
	Adjusted^†^ OR	95%CI	P value	Adjusted^†^ OR	95%CI	P value
Age group						
< 45	1.806	0.459–7.109	0.398	4.136	1.733–9.874	0.001
45–54	1.298	0.484–3.481	0.605	2.758	1.434–5.305	0.002
55–64	0.763	0.343–1.701	0.509	1.769	1.009–3.102	0.046
65–74	1.123	0.548–2.301	0.752	1.867	1.100-3.168	0.021
≥ 75	Reference	Reference		Reference	Reference	
Sex						
Male	1.139	0.586–2.212	0.702	1.976	1.261–3.096	0.003
Female	Reference	Reference		Reference	Reference	
Ethnicity						
Han ethnicity	4.586	0.804–26.168	0.087	1.565	0.803–3.050	0.189
Other	Reference	Reference		Reference	Reference	
Education						
Primary school and preschool	0.812	0.177–3.725	0.789	1.680	0.575–4.908	0.343
Middle school	0.850	0.188–3.830	0.832	1.576	0.550–4.518	0.397
College and higher	Reference	Reference		Reference	Reference	
Marital status						
Single	0.821	0.108–6.263	0.849	0.279	0.037–2.082	0.213
Widowed	0.698	0.317–1.538	0.372	1.326	0.850–2.069	0.214
Other	10.681	1.500-76.031	0.018	0.716	0.065–7.854	0.784
Married	Reference	Reference		Reference	Reference	
Occupation						
Worker	0.413	0.093–1.830	0.244	1.138	0.582–2.222	0.706
Employee	0.630	0.079–5.055	0.664	1.655	0.618–4.433	0.316
Retiree or homemaker	0.791	0.395–1.584	0.508	1.453	0.951–2.220	0.084
Other	1.545	0.221–10.814	0.661	1.057	0.198–5.652	0.948
Farmer	Reference	Reference		Reference	Reference	
Geographic location						
Eastern China	1.087	0.454–2.604	0.851	0.513	0.328–0.802	0.003
Central China	1.471	0.667–3.240	0.339	0.538	0.360–0.806	0.003
Western China	Reference	Reference		Reference	Reference	
Place of residence						
Urban	1.185	0.641–2.191	0.588	1.200	0.820–1.756	0.347
Rural	Reference	Reference		Reference	Reference	
Subtype of first-ever stroke						
Unclassified stroke	1.873	0.436–8.056	0.399	1.321	0.407–4.287	0.643
Subarachnoid hemorrhage	0.351	0.047–2.633	0.309	2.042	1.096–3.805	0.025
Intracerebral hemorrhage	1.213	0.616–2.387	0.577	1.904	1.297–2.795	0.001
Ischemic stroke	Reference	Reference		Reference	Reference	
Disease history						
Hypertension						
Yes	1.114	0.559–2.218	0.760	0.727	0.494–1.068	0.104
No	Reference	Reference		Reference	Reference	
Diabetes mellitus						
Unknown	2.190	1.040–4.613	0.039	0.750	0.383–1.467	0.401
Yes	1.185	0.559–2.512	0.658	0.900	0.549–1.474	0.675
No	Reference	Reference		Reference	Reference	
Dyslipidemia						
Unknown	0.692	0.339–1.412	0.311	0.613	0.378–0.993	0.047
Yes	0.926	0.481–1.780	0.817	1.187	0.803–1.753	0.390
No	Reference	Reference		Reference	Reference	
Atrial fibrillation						
Unknown	0.905	0.346–2.367	0.838	0.838	0.427–1.643	0.607
Yes	0.936	0.215–4.069	0.929	1.147	0.410–3.208	0.794
No	Reference	Reference		Reference	Reference	
Coronary heart disease						
Unknown	1.284	0.506–3.261	0.599	1.242	0.688–2.244	0.472
Yes	1.295	0.670–2.504	0.442	0.722	0.430–1.214	0.219
No	Reference	Reference		Reference	Reference	
Smoking						
Unknown	0.285	0.000-1143.72	0.767	0.746	0.012–47.380	0.890
Regular smoking	0.772	0.324–1.840	0.559	1.409	0.821–2.418	0.213
Occasional smoking	0.637	0.226–1.797	0.394	0.940	0.484–1.827	0.855
Quit smoking	0.601	0.242–1.494	0.273	1.055	0.599–1.856	0.854
Never smoked	Reference	Reference		Reference	Reference	
Alcohol consumption						
Unknown	0.523	0.000-2103.22	0.878	1.886	0.029-120.643	0.765
Regular alcohol consumption	0.769	0.200-2.963	0.703	1.027	0.529–1.995	0.936
Occasional alcohol consumption	1.705	0.753–3.860	0.200	1.166	0.686–1.979	0.571
stopped consuming alcohol	1.644	0.686–3.938	0.265	1.339	0.796–2.252	0.271
Never consumed alcohol	Reference	Reference		Reference	Reference	

†, for each predictor of interest, all other variables in the table were adjusted in a multinomial Logistic regression model

## Discussion

To the best of our knowledge, this is the first large-scale sampling survey on the prevalence of comorbid stroke and TBI in a real-world natural population. The prevalence of comorbid stroke and TBI in the population was not high compared with the higher prevalences of stroke and TBI. However, the rate of post-TBI stroke in patients with previous TBI was up to 11 times higher than the rate of post-stroke TBI in patients with previous stroke, and a marked difference in comorbidities between patients with different subtypes of stroke or TBI existed.

A higher prevalence of comorbid stroke and TBI in the population may be associated with a higher prevalence of stroke and TBI per se and suboptimal management of stroke and TBI patients. On the one hand, previous TBI increased the risk of stroke and its major subtypes in the population in this study (see Supplementary Table 1). Similar to our findings in this study, previous case-control or cohort studies from hospital or insurance data also confirmed that previous TBI, even a concussion, increased the odds ratio or incidence of stroke and its subtype [6, 7, 9, 12–17] and was more closely related to haemorrhagic stroke, including intracerebral haemorrhage and subarachnoid haemorrhage. [6, 7, 9, 16, 17] On the other hand, previous stroke also increased the risk of nonconcussion TBI in the population in this survey (see Supplementary Table 2). Regrettably, despite the higher estimated incidence rates of falls and fall-related injuries of 88.0 and 2.8 per 100 person-years in stroke patients, respectively [18], there is little evidence that stroke increases the risk of TBI. Nonetheless, a retrospective cohort study based on insurance data from Taiwan confirmed that stroke patients had an increased risk of TBI and in-hospital mortality after TBI. [8].

For the first time, this survey has shown that the comorbidity prevalence of post-TBI stroke in patients with previous TBI is up to 11 times higher than that of post-stroke TBI in patients with previous stroke. It is postulated to be due to three reasons: first, the prevalence of stroke is generally higher than that of TBI in the population [1–5]. This was also confirmed by the findings in this survey. Second, the survival time of patients after TBI is generally higher than that of patients after stroke. This can better explain why the median time of stroke after TBI is higher than that of TBI after stroke. Third, ample evidence supports the increased risk of stroke after TBI [6, 7, 9, 12–17].

In this study, the rate of comorbid nonconcussion TBI and stroke among TBI patients was higher than that of comorbid cerebral concussion and stroke. Indeed, a previous study confirmed that higher TBI severity was associated with an increased risk of ischaemic stroke compared to lower TBI severity in older adults with TBI. [19] The rates of post-TBI SAH and post-TBI ICH among stroke patients were also higher than those of post-TBI IS, which was partially supported by previous findings that TBI is more closely related to intracerebral haemorrhage and subarachnoid haemorrhage [6, 7, 9, 16, 17]. In addition, there was a different rate of stroke and TBI comorbidity among stroke or TBI patients by different age groups and geographic locations; however, the true reasons underlying this are unclear.

In this survey, we did not find a difference in the rate of pre-TBI stroke or post-TBI stroke among stroke patients between the two groups with and without any of the included stroke-related factors, implying no difference in care for comorbid stroke and TBI between the groups. Regrettably, because data on these risk factors were not collected for all people who participated in the survey, further analyses on associations between stroke-related risk factors and comorbid stroke and TBI in the population were limited.

Previous findings suggest that stroke risk may be highest in the first four months post-TBI but remains significant up to five years post-TBI. [6] However, the median time to stroke onset post-TBI was 11.07 (IQR: 2.97–26.98) years among 158 post-TBI stroke survivors in this study, implying that the prevention of stroke after TBI should be unremitting for a long time. The increased long-term risk for stroke following TBI was further confirmed by a study of veterans with prior TBI [12].

Our findings implied that the high prevalence of stroke among TBI patients is becoming a new public health issue. In this survey, the stroke prevalence after TBI in the real-world natural population was significantly higher than that in the general population. Given the large number of TBI patients in the population [3, 5], as well as previous prospective studies confirming that TBI significantly increases the risk of post-TBI stroke [6, 7, 9, 12–17] and the insufficient attention given to the increasing risk of stroke in TBI patients, these findings can be well understood. The findings in this real-world cross-sectional survey suggest that early comorbid risk warning and timely intervention are needed for a large number of TBI patients to effectively prevent and control stroke after TBI in the population. The same reasons can explain why the prevalence of TBI after stroke in the real-world population is higher than that in the general population [1, 2, 4, 8]; early comorbid risk warning and timely intervention measures can also be implemented for stroke patients. Early comorbid risk warning and timely intervention for stroke and TBI patients have significant practical significance not only for the primary prevention of stroke and TBI per se but also for the prevention and control of comorbid stroke and TBI. Clinical physicians in hospitals and primary health care staff in community health service centres should pay attention not only to the primary prevention of stroke and TBI in the population but also to comorbidity development in patients with stroke or TBI.

Our study with a very large sample size and an 81% participation rate has better national representativeness in China, and standardized methodology and diagnostic criteria were assured in this survey. However, our study has several limitations. First, recall bias may have resulted in underestimation of the prevalence of stroke, TBI, and their comorbidity, especially for mild stroke or TBI, in this cross-sectional study. The recall bias may have been minimized by our cross-checking of the data obtained during the door-to-door interviews with those from medical records. Nearly 10% of cases of stroke and 40% of cases of TBI were not confirmed with CT/MRI imaging in this survey. However, all individuals with suspected stroke or TBI were further interviewed by trained neurologists, further enhancing the accuracy of diagnosis and the dates of stroke and TBI events. Second, the nonresponse rate was 19%, slightly greater than that of 15% that we accounted for in the sample size calculation. However, this potentially would not have largely affected our results given that the socio-demographic characteristics of nonrespondents were not significantly different from those who were interviewed for the study. Third, TBI case ascertainment was based on medical records from hospitals of different grades as well as injury history. Considering the feasibility of the population, we could not collect data on Glasgow Coma Scale (GCS) scores at the time of an injury, especially for patients not accessing the hospital. Accordingly, we cannot present the classification of TBI severity according to GCS scores.

Our findings in this survey should have better generalizability because the majority of the findings in the real-world natural population have been confirmed by previous similar studies, whether in the Chinese population [6, 9, 14, 15, 17] or the US population [6, 12, 13, 16]. In this survey, the prevalence of stroke was higher than that of TBI, and TBI patients, especially nonconcussion TBI patients, were more likely to develop comorbid stroke and TBI than stroke patients, especially ischaemic stroke patients. Comparing the prevalence of stroke and TBI across various countries or regions, previous studies have confirmed that the former is generally higher than the latter [1–5]. Previous studies also confirmed that previous TBI, even a concussion, increased the risk of stroke and its subtypes [6, 7, 9, 12–17] and was more closely related to haemorrhagic stroke. [6, 7, 9, 16, 17] A retrospective cohort study also confirmed that stroke patients had an increased risk of TBI and in-hospital mortality after TBI. [8] Nonetheless, considering the research limitations mentioned above, the findings should be interpreted with caution when extrapolated to other populations.

Regarding the increase with age and male preponderance of comorbid stroke and TBI in this survey, we did not find any comparable data after reviewing relevant documents. Given that stroke and TBI increase each other’s risk, it remains to be further confirmed whether early comorbid risk warning and timely intervention for stroke and TBI patients can effectively prevent and control comorbid stroke and TBI. In the future, more research is expected to conduct surveys on the prevalence of comorbid stroke and TBI in other countries or regions to guide the prevention and control of comorbid stroke and TBI in the local population, as well as to evaluate the effectiveness of early comorbid risk warning and timely intervention for relevant professionals and patients. This was a cross-sectional survey, and it is difficult to determine the true causality for the increased risk of comorbidity following stroke and TBI. The mechanisms underlying comorbid stroke and TBI also need further clarification in future research.

## Conclusion

In conclusion, the high prevalence of stroke among TBI patients is becoming a new public health issue. TBI patients, especially nonconcussion TBI patients, are more likely to develop comorbid stroke and TBI than stroke patients, especially ischaemic stroke patients. However, it is worth noting that the burden of stroke and TBI comorbidity in the future will be expected to rise with an increasing trend of the prevalence of stroke and TBI per se, as well as suboptimal prevention and control of patients with previous stroke or TBI. The major findings in this study can not only enable evidence-based health care planning for the prevention and control of comorbid stroke and TBI but also help physicians and patients with previous stroke or TBI in the population better understand and care for the prevention of comorbidity with the two diseases.

### Electronic supplementary material

Below is the link to the electronic supplementary material.

Supplementary Material 1

Supplementary Material 2

## Data Availability

All data relevant to the study are included in the article or uploaded as supplementary information. No additional data are available.

## List of abbreviations

TBI: Traumatic brain injury
PPS: Probability proportionate to population size
DSPs: Disease surveillance points
CDC: Centers for Disease Control and Prevention
SAH: Subarachnoid hemorrhage
ICH: Intracerebral hemorrhage
IS: Ischemic stroke
CHD: Coronary heart disease
WHO: World Health Organization
IQR: Interquartile range
OR: Odds ratio
CI: Confidence interval
GCS: Glasgow Coma Scale
