# Supplementary material for: Prevalence and characteristics of comorbid stroke and traumatic brain injury in a real-world population: findings from a nationally representative cross-sectional survey in China
Source: BMC Public Health. 2023 Oct 18;23:2037. doi: 10.1186/s12889-023-16990-0 (PMC10585840; doi:10.1186/s12889-023-16990-0)
Supplement: Supplementary file 1 — Supplementary Material 1 [file 12889_2023_16990_MOESM1_ESM.docx]

| **Supplementary Table 1 Prevalence (1/100,000 person*lifetime) and rate ratio of stroke among different subgroups of the Chinese population** | | | | | | | | | | | | | | | | | | |  |
| --- | --- | --- | --- | --- | --- | --- | --- | --- | --- | --- | --- | --- | --- | --- | --- | --- | --- | --- | --- |
|  |  | Stroke prevalence | |  | |  |  | IS prevalence | |  | |  | |  | Undetermined Stroke prevalence | |  | | |
| Factors | Rate (95%CI) | | Rate ratio^†^ (95%CI) | | P value |  | Rate (95%CI) | | Rate ratio^†^ (95%CI) | | P value | |  | Rate (95%CI) | | Rate ratio^†^ (95%CI) | | P value |  |
| Age group |  | |  | |  |  |  | |  | |  | |  |  | |  | |  |  |
| 0~ | 6.02  (1.96-14.05) | | 0.001 (0.000-0.003) | | <0.001 |  | 1.20  (0.03-6.71) | | <0.001 (<0.001-0.002) | | <0.001 | |  | - | | - | | - |  |
| 15~ | 5.17  (1.41-13.24) | | 0.001 (0.000-0.003) | | <0.001 |  | - | | - | | - | |  | - | | - | | - |  |
| 25~ | 38.28  (26.66-53.24) | | 0.007 (0.005-0.010) | | <0.001 |  | 24.06  (15.08-36.43) | | 0.005  (0.003-0.008) | | <0.001 | |  | - | | - | | - |  |
| 35~ | 148.62 (125.64-174.59) | | 0.027 (0.022-0.033) | | <0.001 |  | 96.40  (78.09-117.73) | | 0.020  (0.016-0.026) | | <0.001 | |  | 6.03  (2.21-13.11) | | 0.046  (0.014-0.152) | | <0.001 |  |
| 45~ | 872.49 (813.71-934.38) | | 0.157 (0.135-0.182) | | <0.001 |  | 604.77  (556.01-656.66) | | 0.126  (0.107-0.148) | | <0.001 | |  | 21.33  (13.03-32.95) | | 0.164  (0.061-0.437) | | <0.001 |  |
| 55~ | 2981.03 (2862.68-3103.02) | | 0.534 (0.466-0.612) | | <0.001 |  | 2264.79  (2161.78-2371.43) | | 0.469  (0.405-0.544) | | <0.001 | |  | 54.90  (39.89-73.71) | | 0.428  (0.170-1.080) | | 0.072 |  |
| 65~ | 5587.04 (5370.33-5810.25) | | 1.007 (0.878-1.154) | | 0.924 |  | 4424.56  (4231.94-4623.69) | | 0.924  (0.797-1.070) | | 0.291 | |  | 95.94  (69.43-129.24) | | 0.753  (0.298-1.902) | | 0.548 |  |
| 75~ | 6719.85 (6383.05-7069.81) | | 1.222 (1.062-1.406) | | 0.005 |  | 5597.62  (5290.60-5917.81) | | 1.178  (1.013-1.371) | | 0.034 | |  | 162.25  (113.64-224.62) | | 1.329  (0.521-3.389) | | 0.551 |  |
| 85~ | 5361.42 (4682.25-6111.41) | | Reference | |  |  | 4643.37  (4012.92-5344.76) | | Reference | |  | |  | 119.67  (38.86-279.28) | | Reference | |  |  |
| Sex |  | |  | |  |  |  | |  | |  | |  |  | |  | |  |  |
| Male | 1394.94 (1353.00-1437.84) | | 1.278 (1.222-1.337) | | <0.001 |  | 1090.76  (1353.72-1128.78) | | 1.302  (1.237-1.370) | | <0.001 | |  | 26.32  (20.84-32.80) | | 1.097  (0.800-1.506) | | 0.565 |  |
| Female | 1157.88 (1119.45-1197.28) | | Reference | |  |  | 892.96  (859.26-927.65) | | Reference | |  | |  | 25.31  (19.91-31.73) | | Reference | |  |  |
| Place of residence |  | |  | |  |  |  | |  | |  | |  |  | |  | |  |  |
| Urban | 1238.18 (1197.51-1279.87) | | 0.810 (0.774-0.847) | | <0.001 |  | 960.00  (924.23-996.80) | | 0.800  (0.760-0.843) | | <0.001 | |  | 15.20  (11.00-20.47) | | 0.392  (0.275-0.559) | | <0.001 |  |
| Rural | 1312.35 (1272.56-1353.07) | | Reference | |  |  | 1021.82  (986.74-1057.82) | | Reference | |  | |  | 35.40  (29.12-42.63) | | Reference | |  |  |
| Geographic Location |  | |  | |  |  |  | |  | |  | |  |  | |  | |  |  |
| Eastern China | 1197.49 (1150.16-1246.26) | | 1.128 (1.056-1.206) | | <0.001 |  | 943.69  (901.73-987.10) | | 1.186  (1.100-1.280) | | <0.001 | |  | 22.35  (16.30-29.91) | | 0.605  (0.404-0.907) | | 0.015 |  |
| Central China | 1594.92 (1544.76-1646.30) | | 1.651 (1.552-1.756) | | <0.001 |  | 1246.99  (1202.68-1292.51) | | 1.729  (1.610-1.856) | | <0.001 | |  | 24.61  (18.74-31.75) | | 0.779  (0.533-1.138) | | 0.197 |  |
| Western China | 890.38 (844.08-938.56) | | Reference | |  |  | 663.28  (623.41-705.04) | | Reference | |  | |  | 32.17  (23.87-42.41) | | Reference | |  |  |
| History of TBI^$^ |  | |  | |  |  |  | |  | |  | |  |  | |  | |  |  |
| Individuals with previous TBI | 6021.34 (5119.06-7036.86) | | 2.468 (2.108-2.889) | | <0.001 |  | 3887.20  (3169.54-4718.79) | | 2.037  (1.674-2.478) | | <0.001 | |  | 114.33  (23.58-334.12) | | 2.250  (0.716-7.071) | | 0.165 |  |
| Individuals without TBI | 1256.21 (1227.86-1285.04) | | Reference | |  |  | 979.71  (954.69-1005.21) | | Reference | |  | |  | 25.43  (21.53-29.82) | | Reference | |  |  |
| †, age group, sex, place of residence, geographic location, and TBI history were introduced in a Poisson regression analysis. For each predictor of interest, all other variables in the table were adjusted in a Poisson regression model. ^$^TBI, traumatic brain injury | | | | | | | | | | | | | | | | | | |  |

| **Supplementary Table 1 Prevalence (1/100,000 person*lifetime) and rate ratio of stroke among different subgroups of the Chinese population (continued)** | | | | | | | | | | | | | | | | | | |  |
| --- | --- | --- | --- | --- | --- | --- | --- | --- | --- | --- | --- | --- | --- | --- | --- | --- | --- | --- | --- |
|  |  | Hemorrhagic Stroke prevalence | |  | |  |  | ICH prevalence | |  | |  | |  | SAH prevalence | |  | | |
| Factors | Rate (95%CI) | | Rate ratio^†^ (95%CI) | | P value |  | Rate (95%CI) | | Rate ratio^†^ (95%CI) | | P value | |  | Rate (95%CI) | | Rate ratio^†^ (95%CI) | | P value |  |
| Age group |  | |  | |  |  |  | |  | |  | |  |  | |  | |  |  |
| 0~ | 4.82  (1.31-12.34) | | 0.008  (0.003-0.022) | | <0.001 |  | 3.61  (0.75-10.56) | | 0.009  (0.003-0.029) | | <0.001 | |  | 1.20  (0.03-6.71) | | 0.006  (0.001-0.050) | | <0.001 |  |
| 15~ | 5.17  (1.41-13.24) | | 0.008  (0.003-0.024) | | <0.001 |  | 5.17  (1.41-13.24) | | 0.012  (0.004-0.037) | | <0.001 | |  |  | |  | | 0.989 |  |
| 25~ | 14.22  (7.57-24.31) | | 0.023  (0.012-0.045) | | <0.001 |  | 12.03  (6.01-21.53) | | 0.029  (0.013-0.061) | | <0.001 | |  | 2.19  (0.26-7.90) | | 0.011  (0.002-0.053) | | <0.001 |  |
| 35~ | 46.19  (33.82-61.61) | | 0.075  (0.046-0.122) | | <0.001 |  | 37.16  (26.16-51.21) | | 0.088  (0.050-0.157) | | <0.001 | |  | 9.04  (4.31-17.87) | | 0.046  (0.018-0.120) | | <0.001 |  |
| 45~ | 246.39  (215.64-280.29) | | 0.396  (0.262-0.599) | | <0.001 |  | 196.26  (168.92-226.75) | | 0.463  (0.282-0.761) | | 0.002 | |  | 50.13  (36.83-66.66) | | 0.254  (0.120-0.538) | | <0.001 |  |
| 55~ | 661.34  (606.23-720.12) | | 1.058  (0.708-1.580) | | 0.783 |  | 541.55  (491.79-594.98) | | 1.272  (0.783-2.065) | | 0.331 | |  | 119.79  (97.03-146.28) | | 0.602  (0.293-1.239) | | 0.169 |  |
| 65~ | 1066.54  (973.05-1166.57) | | 1.704  (1.140-2.548) | | 0.009 |  | 818.87  (737.22-907.08) | | 0.921  (1.181-3.125) | | 0.009 | |  | 247.67  (203.74-298.26) | | 1.243  (0.606-2.548) | | 0.553 |  |
| 75~ | 959.98  (835.38-1097.92) | | 1.549  (1.024-2.345) | | 0.038 |  | 676.04  (572.18-793.30) | | 1.603  (0.971-2.648) | | 0.065 | |  | 283.94  (218.19-363.28) | | 1.436  (0.688-2.998) | | 0.335 |  |
| 85~ | 598.37  (387.24-883.32) | | Reference | |  |  | 406.89  (237.03-651.48) | | Reference | |  | |  | 191.48  (82.67-377.29) | | Reference | |  |  |
| Sex |  | |  | |  |  |  | |  | |  | |  |  | |  | |  |  |
| Male | 277.85  (259.31-297.37) | | 1.210  (1.095-1.338) | | <0.001 |  | 220.88  (204.39-238.36) | | 1.254  (1.119-1.405) | | <0.001 | |  | 56.97  (48.75-66.18) | | 1.068  (0.862-1.322) | | 0.549 |  |
| Female | 239.61  (222.30-257.90) | | Reference | |  |  | 183.59  (168.48-199.68) | | Reference | |  | |  | 56.02  (47.82-65.22) | | Reference | |  |  |
| Place of residence |  | |  | |  |  |  | |  | |  | |  |  | |  | |  |  |
| Urban | 262.98  (244.42-282.57) | | 0.904  (0.817-1.000) | | 0.049 |  | 204.30  (187.98-221.66) | | 0.902  (0.805-1.011) | | 0.075 | |  | 58.67  (50.09-68.31) | | 0.911  (0.735-1.130) | | 0.397 |  |
| Rural | 255.14  (237.76-273.45) | | Reference | |  |  | 200.60  (185.23-216.91) | | Reference | |  | |  | 54.54  (46.67-63.35) | | Reference | |  |  |
| Geographic Location |  | |  | |  |  |  | |  | |  | |  |  | |  | |  |  |
| Eastern China | 231.45  (210.91-253.45) | | 1.016  (0.879-1.175) | | 0.829 |  | 183.77  (165.52-203.48) | | 1.003  (0.853-1.179) | | 0.973 | |  | 47.68  (38.62-58.23) | | 1.071  (0.774-1.483) | | 0.678 |  |
| Central China | 323.32  (300.96-346.91) | | 1.526  (1.335-1.745) | | <0.001 |  | 247.39  (227.88-268.13) | | 1.451  (1.249-1.687) | | <0.001 | |  | 75.93  (65.30-87.80) | | 1.836  (1.366-2.468) | | <0.001 |  |
| Western China | 194.93  (173.60-218.16) | | Reference | |  |  | 156.97  (137.89-177.96) | | Reference | |  | |  | 37.96  (28.89-48.96) | | Reference | |  |  |
| History of TBI^$^ |  | |  | |  |  |  | |  | |  | |  |  | |  | |  |  |
| Individuals with previous TBI | 2019.82  (1512.98-2641.97) | | 4.200  (3.191-5.528) | | <0.001 |  | 1562.50  (1121.28-2119.71) | | 4.136  (3.027-5.652) | | <0.001 | |  | 457.32  (236.30-798.84) | | 4.431  (2.487-7.896) | | <0.001 |  |
| Individuals without TBI | 251.07  (238.49-264.15) | | Reference | |  |  | 196.35  (185.24-207.95) | | Reference | |  | |  | 54.73  (48.94-61.01) | | Reference | |  |  |
| †, age group, sex, place of residence, geographic location, and TBI history were introduced in a Poisson regression analysis. For each predictor of interest, all other variables in the table were adjusted in a Poisson regression model. ^$^TBI, traumatic brain injury | | | | | | | | | | | | | | | | | | |  |
