# Supplementary material for: Prevalence and characteristics of comorbid stroke and traumatic brain injury in a real-world population: findings from a nationally representative cross-sectional survey in China
Source: BMC Public Health. 2023 Oct 18;23:2037. doi: 10.1186/s12889-023-16990-0 (PMC10585840; doi:10.1186/s12889-023-16990-0)
Supplement: Supplementary file 2 — Supplementary Material 2 [file 12889_2023_16990_MOESM2_ESM.docx]

| **Supplementary Table 2 Prevalence (1/100,000 person*lifetime) and rate ratio of traumatic brain injury (TBI) among different subgroups of the Chinese population** | | | | | | | | | | | |
| --- | --- | --- | --- | --- | --- | --- | --- | --- | --- | --- | --- |
|  |  | TBI prevalence |  |  |  | Concussion prevalence |  |  |  | Nonconcussion prevalence |  |
| Factors | Rate (95%CI) | Rate ratio^†^ (95%CI) | P value |  | Rate (95%CI) | Rate ratio^†^ (95%CI) | P value |  | Rate (95%CI) | Rate ratio^†^ (95%CI) | P value |
| Age group |  |  |  |  |  |  |  |  |  |  |  |
| 0~ | 63.83 (47.82-83.50) | 0.140  (0.080-0.244) | <0.001 |  | 34.93  (23.39-50.16) | 0.131  (0.062-0.277) | <0.001 |  | 28.91  (18.52-43.01) | 0.156  (0.067-0.363) | <0.001 |
| 15~ | 118.94 (95.88-145.86) | 0.259 (0.152-0.440) | <0.001 |  | 71.10  (53.56-92.55) | 0.265  (0.131-0.536) | <0.001 |  | 47.83  (33.68-65.93) | 0.259  (0.115-0.581) | 0.001 |
| 25~ | 204.52 (176.26-236.03) | 0.472 (0.283-0.788) | 0.004 |  | 136.71  (113.80-162.88) | 0.541  (0.275-1.065) | 0.075 |  | 67.81  (51.99-86.93) | 0.387  (0.177-0.847) | 0.017 |
| 35~ | 368.57 (331.82-408.27) | 0.826 (0.500-1.362) | 0.453 |  | 257.09  (226.56-290.59) | 0.987  (0.508-1.920) | 0.970 |  | 111.47  (91.70-134.24) | 0.618  (0.288-1.329) | 0.218 |
| 45~ | 610.22 (561.23-662.34) | 1.412 (0.859-2.321) | 0.174 |  | 433.13  (392.02-477.38) | 1.731  (0.894-3.351) | 0.104 |  | 177.09  (151.18-206.17) | 0.998  (0.468-2.129) | 0.997 |
| 55~ | 833.87 (771.83-899.58) | 1.999 (1.218-3.283) | 0.006 |  | 585.46  (533.67-640.92) | 2.463  (1.273-4.764) | 0.007 |  | 248.41  (215.10-285.43) | 1.400  (0.659-2.975) | 0.382 |
| 65~ | 861.91 (778.06-952.34) | 2.002 (1.214-3.301) | 0.007 |  | 578.33  (510.04-653.23) | 2.386  (1.227-4.637) | 0.010 |  | 283.58  (236.41-337.41) | 1.509  (0.705-3.231) | 0.289 |
| 75~ | 838.52 (722.34-968.07) | 2.029 (1.218-3.382) | 0.007 |  | 554.5  (460.85-661.60) | 2.405  (1.222-4.732) | 0.011 |  | 284.01  (218.24-363.38) | 1.543  (0.706-3.369) | 0.277 |
| 85~ | 383.05 (218.95-622.05) | Reference |  |  | 215.47  (98.52-409.02) | Reference |  |  | 167.58  (67.38-345.29) | Reference |  |
| Sex |  |  |  |  |  |  |  |  |  |  |  |
| Male | 534.53 (508.69-561.34) | 1.769 (1.632-1.919) | <0.001 |  | 352.24  (331.33-374.14) | 1.603  (1.455-1.765) | <0.001 |  | 182.29  (167.33-198.22) | 2.219  (1.910-2.578) | <0.001 |
| Female | 311.51 (291.73-332.27) | Reference |  |  | 227.47  (210.62-245.31) | Reference |  |  | 84.04  (73.92-95.15) | Reference |  |
| Place of residence |  |  |  |  |  |  |  |  |  |  |  |
| Urban | 420.70 (397.14-445.30) | 0.983 (0.909-1.064) | 0.671 |  | 292.02  (272.44-312.63) | 1.009  (0.918-1.110) | 0.846 |  | 128.69  (115.80-142.61) | 0.928  (0.806-1.068) | 0.298 |
| Rural | 426.45 (403.89-449.94) | Reference |  |  | 288.66  (270.16-308.09) | Reference |  |  | 137.79  (125.10-151.42) | Reference |  |
| Geographic Location |  |  |  |  |  |  |  |  |  |  |  |
| Eastern China | 273.21 (250.85-297.02) | 0.384 (0.346-0.427) | <0.001 |  | 173.86  (156.12-193.06) | 0.341  (0.300-0.388) | <0.001 |  | 99.35  (86.06-114.11) | 0.493  (0.411--0.592) | <0.001 |
| Central China | 412.66 (387.34-439.21) | 0.608 (0.556-0.665) | <0.001 |  | 281.65  (260.80-303.72) | 0.582  (0.523-0.647) | <0.001 |  | 131.02  (116.93-146.34) | 0.674  (0.573-0.793) | <0.001 |
| Western China | 635.76 (596.73-676.67) | Reference |  |  | 454.30  (421.40-489.08) | Reference |  |  | 181.46  (160.90-203.93) | Reference |  |
| History of stroke |  |  |  |  |  |  |  |  |  |  |  |
| Individuals with previous stroke | 811.06 (620.40-1041.84) | 1.005 (0.777-1.300) | 0.969 |  | 212.74  (121.60-345.47) | 0.379  (0.231-0.621) | <0.001 |  | 598.32  (436.42-800.61) | 2.441  (1.791-3.327) | <0.001 |
| Individuals without stroke | 418.78 (402.41-435.64) | Reference |  |  | 291.24  (277.62-305.36) | Reference |  |  | 127.54  (118.58-136.99) | Reference |  |
| †, age group, sex, place of residence, geographic location, and stroke history were introduced in a Poisson regression analysis. For each predictor of interest, all other variables in the table were adjusted in a Poisson regression model. | | | | | | | | | | | |
